# Comparative clinical outcomes of SMILE, femtosecond LASIK, and transepithelial PRK: a multicenter Iraqi study

**DOI:** 10.3389/fopht.2026.1787176

**Published:** 2026-04-14

**Authors:** Hassan A. Aljaberi, Saeed Rahmani, Humam H. Alrikabi

**Affiliations:** 1 Optical Techniques Department, College of Health and Medical Techniques, Al-Mustaqbal University, Babylon, Iraq; 2 Department of Optometry, Faculty of Rehabilitation, Shahid Beheshti University of Medical Sciences, Tehran, Iran

**Keywords:** FEMTOLASIK, higher-order aberrations, myopia, ocular surface disease index, small incision lenticule extraction (SMILE), transepithelial photorefractive keratectomy

## Abstract

**Purpose:**

To compare refractive predictability, long-term stability, visual quality, ocular surface outcomes, and safety of small incision lenticule extraction (SMILE), femtosecond laser–assisted laser *in situ* keratomileusis (FS-LASIK), and transepithelial photorefractive keratectomy (Trans-PRK) for myopia correction in an Iraqi population.

**Methods:**

This retrospective multicenter comparative cohort study included 919 eyes of 919 patients who underwent SMILE (388 eyes), FS-LASIK (344 eyes), or Trans-PRK (187 eyes) between January 2023 and December 2025. Postoperative outcomes were evaluated at 6 months, 1 year, and 1.5 years. Primary outcome measures included refractive predictability and stability of spherical equivalent (SE). Secondary outcomes included uncorrected and corrected distance visual acuity (UDVA and CDVA), absolute refractive error, induced corneal higher-order aberrations (HOAs) at 6 months, Ocular Surface Disease Index (OSDI) scores, and safety outcomes. Multivariable linear regression analysis was performed to identify factors associated with residual SE at 1.5 years.

**Results:**

SMILE demonstrated the highest refractive predictability and long-term stability, with postoperative SE values closest to emmetropia and the narrowest distribution of residual refractive error at all follow-up intervals. FS-LASIK showed intermediate outcomes, whereas Trans-PRK was associated with greater residual myopia and increased refractive regression over time. Induced corneal HOAs were lowest following SMILE and highest following Trans-PRK. OSDI scores were consistently lower after SMILE, intermediate after FS-LASIK, and highest after Trans-PRK throughout follow-up. Multivariable analysis identified surgical technique as the primary independent predictor of residual SE at 1.5 years, with FS-LASIK and Trans-PRK associated with significantly greater myopic residual error than SMILE. All three procedures demonstrated a high safety profile, with low rates of CDVA loss and infrequent enhancement procedures.

**Conclusions:**

In this large multicenter Iraqi cohort, SMILE provided superior refractive predictability, long-term stability, visual quality, and ocular surface outcomes compared with FS-LASIK and Trans-PRK. FS-LASIK remained an effective and safe alternative, while Trans-PRK was associated with greater refractive regression and higher enhancement rates, particularly in eyes with higher degrees of myopia. These findings support procedure-specific patient selection to optimize refractive outcomes and postoperative comfort.

## Introduction

Myopia is among the most prevalent refractive errors worldwide and represents a growing public health concern due to its increasing prevalence and association with potentially sight-threatening ocular complications. Epidemiological studies have documented a marked rise in the global prevalence of myopia and high myopia over recent decades, with projections suggesting that nearly half of the world’s population may be affected by myopia by 2050 (1–3). High myopia is associated with an increased risk of retinal degeneration, myopic maculopathy, glaucoma, and irreversible visual impairment, underscoring the importance of effective, predictable, and safe refractive correction strategies (4–6).

Laser refractive surgery has become an established modality for correcting myopia and has evolved substantially with advances in corneal laser technology. Early surface ablation procedures, such as photorefractive keratectomy (PRK), formed the foundation of corneal refractive surgery and were followed by the widespread adoption of laser-assisted *in situ* keratomileusis (LASIK). The introduction of femtosecond laser technology led to the development of femtosecond laser–assisted LASIK (FS-LASIK), which improved flap reproducibility, refractive accuracy, and safety profiles. More recently, minimally invasive flapless techniques, particularly small incision lenticule extraction (SMILE), a form of keratorefractive lenticule extraction (KLEx), along with refinements in surface ablation methods such as transepithelial PRK (Trans-PRK), have expanded the spectrum of surgical options available for myopia correction (7–9).

FS-LASIK remains one of the most commonly performed refractive procedures globally. The creation of a femtosecond laser–generated corneal flap, followed by excimer laser stromal ablation, enables rapid visual rehabilitation, high refractive predictability, and excellent patient-reported satisfaction (10–12). However, corneal flap creation alters anterior corneal biomechanics and has been associated with postoperative dry eye symptoms and rare flap-related complications, including epithelial ingrowth and flap displacement (13–16). These limitations have driven growing interest in flapless refractive surgical techniques.

SMILE represents a distinct flapless surgical approach in which a refractive lenticule is created within the corneal stroma and extracted through a small incision, thereby preserving the anterior corneal lamellae and subbasal nerve plexus (8, 17). Several studies have demonstrated that SMILE provides visual and refractive outcomes comparable to those of FS-LASIK, while potentially offering advantages in corneal biomechanical stability and reduced early postoperative dry eye symptoms (18–20). Nevertheless, early visual recovery following SMILE may be slightly delayed, and enhancement strategies can be more technically demanding compared with flap-based procedures (21).

Trans-PRK is a modern surface ablation technique that enables the removal of epithelium and stromal ablation in a single-step excimer laser procedure, eliminating the need for mechanical or alcohol-assisted epithelial debridement. By avoiding corneal flap creation, Trans-PRK preserves corneal biomechanical integrity and eliminates flap-related complications, making it particularly suitable for patients with thin corneas, borderline corneal topography, or an increased risk of corneal ectasia (22–25). Despite these advantages, Trans-PRK is commonly associated with greater early postoperative discomfort, slower visual recovery, and a potential risk of postoperative corneal haze, particularly in eyes with high myopia (26).

Comparative studies evaluating FS-LASIK, SMILE, and PRK-based techniques have generally reported high efficacy and safety for myopia correction, with excellent uncorrected distance visual acuity and refractive predictability across procedures (27). Direct comparisons between FS-LASIK and SMILE have demonstrated largely comparable visual and refractive outcomes, although differences in higher-order aberrations, contrast sensitivity, and ocular surface parameters have been reported (28, 29). Studies assessing Trans-PRK suggest superior preservation of corneal biomechanics compared with flap-based techniques, albeit at the expense of slower visual rehabilitation (30). Despite these findings, reported outcomes remain heterogeneous owing to differences in patient selection, refractive error magnitude, surgical platforms, and follow-up duration.

Most comparative refractive surgery studies have been conducted in East Asian, European, or North American populations, whereas data from Middle Eastern populations, including Iraqi patients, remain limited. Ethnic, regional, and environmental factors may influence corneal morphology, wound-healing responses, and postoperative outcomes, highlighting the need for population-specific comparative data. Furthermore, many previous studies have focused primarily on refractive accuracy and visual acuity, placing less emphasis on a comprehensive assessment of visual quality and ocular surface outcomes.

The present study aimed to compare refractive predictability, visual acuity, visual quality as assessed by higher-order aberrations, and ocular surface outcomes following SMILE, FS-LASIK, and Trans-PRK for myopia correction in a large cohort of Iraqi patients. It was hypothesized that flapless refractive techniques would demonstrate superior ocular surface outcomes while maintaining refractive accuracy and visual acuity comparable to those of flap-based procedures.

## Materials and methods

### Study design and setting

This retrospective, multicenter, comparative cohort study evaluated refractive, visual, visual quality, and ocular surface outcomes following three contemporary laser refractive surgery techniques for myopia correction: small incision lenticule extraction (SMILE), femtosecond laser–assisted LASIK (FS-LASIK), and transepithelial PRK (Trans-PRK). Medical records of patients who underwent laser refractive surgery between January 2023 and December 2025 were reviewed at three tertiary eye care centers in Iraq. The study was designed and reported in accordance with the Strengthening the Reporting of Observational Studies in Epidemiology (STROBE) guidelines.

Patients aged 22 to 35 years who underwent laser refractive surgery for myopia with or without astigmatism were eligible for inclusion. All included eyes demonstrated documented refractive stability for at least 12 months before surgery. To minimize inter-eye correlation and statistical dependency, only the right eye of each patient was included in the analysis.

A total of 919 eyes met the eligibility criteria and were included in the final analysis. All patients completed at least 6 months of postoperative follow-up, with additional follow-up data available at 1 year and 1.5 years. A formal sample size calculation was not performed due to the retrospective nature of the study; however, all eligible cases during the study period were included to maximize statistical power and external validity.

### Inclusion and exclusion criteria

Eyes were included if preoperative corrected distance visual acuity (CDVA) was 20/25 or better and no ocular pathology was present that could adversely affect refractive or visual quality outcomes. Exclusion criteria comprised previous ocular surgery, corneal ectatic disorders or suspected keratoconus, ocular trauma, active ocular surface disease, retinal pathology, glaucoma, systemic diseases known to impair corneal wound healing, incomplete clinical records, or insufficient postoperative follow-up.

### Study centers and surgical distribution

Clinical data were collected from Ibn Al-Haitham Teaching Eye Hospital, Al-Hakeem Teaching Hospital, and Imam Hussain Specialized Eye Complex, Faculty of Medicine. Selection of the refractive surgical technique was based on corneal morphology, refractive error magnitude, and overall clinical suitability as determined by the treating surgeon at each center. As surgical allocation was non-randomized, potential selection bias was addressed through multivariable regression analyses adjusting for relevant baseline covariates.

Eyes were stratified according to the refractive procedure performed into three groups: SMILE (388 eyes), FS-LASIK (344 eyes), and Trans-PRK (187 eyes).

### Preoperative assessment

All patients underwent a comprehensive preoperative ophthalmic evaluation, including uncorrected distance visual acuity (UDVA), corrected distance visual acuity (CDVA), manifest refraction, slit-lamp biomicroscopy, and dilated fundus examination. Spherical equivalent (SE) was calculated as the spherical component plus half the cylindrical component, with astigmatism defined as the absolute value of the cylindrical component.

Corneal assessment included keratometry, central corneal thickness (CCT) measurement, and corneal tomography to evaluate corneal shape and surgical suitability. Mesopic pupil diameter was recorded when available. Based on preoperative spherical equivalent, eyes were categorized as having mild (≤ −3.00 diopters), moderate (−3.01 to −6.00 diopters), or high (> −6.00 diopters) myopia.

### Laser systems and surgical platforms

Small-incision lenticule extraction procedures were performed using a femtosecond laser platform (VisuMax, Carl Zeiss Meditec, Jena, Germany) or equivalent systems with comparable technical specifications available at the participating centers.

Femtosecond laser–assisted LASIK procedures involved femtosecond laser flap creation using platforms manufactured by Alcon Laboratories (Fort Worth, TX, USA) or NIDEK Co., Ltd. (Gamagori, Japan), followed by excimer laser stromal ablation using corresponding excimer laser systems from the same manufacturers. Wavefront-optimized or wavefront-guided ablation profiles were selected based on corneal characteristics and surgeon preference at each center.

Transepithelial photorefractive keratectomy procedures were performed using excimer laser platforms from Alcon or NIDEK capable of single-step epithelial removal and stromal ablation. Optical zone and transition zone parameters were individualized according to preoperative refractive error, corneal thickness, and corneal curvature. Mitomycin C (0.02%) was applied selectively in eyes undergoing higher myopic correction, followed by copious irrigation.

All laser systems were routinely calibrated and maintained in accordance with manufacturers’ specifications. Surgical parameters were standardized as much as possible across centers to minimize inter-platform variability.

Laser treatment planning was not based solely on manifest refraction. Instead, individualized adjustments were applied using surgeon-specific nomograms derived from device-specific recommendations and clinical experience at each participating center. Optical zone, transition zone, and femtosecond-laser parameters were selected within clinically accepted ranges and tailored to corneal characteristics and refractive error. Although efforts were made to standardize surgical approaches across centers, some variability in nomogram optimization may have occurred, which was taken into account when interpreting the results.

### Postoperative follow-up

Postoperative examinations were conducted at 6 months post-surgery, with additional follow-up at 1 and 1.5 years. At each visit, UDVA, CDVA, manifest refraction, and spherical equivalent were recorded. Postoperative complications, adverse events, and enhancement (retreatment) procedures were documented throughout the follow-up period.

All included patients completed follow-up through 1.5 years, and no cases were excluded due to incomplete follow-up.

### Visual quality and ocular surface assessment

Visual quality was assessed by measuring corneal higher-order aberrations (HOAs), including total higher-order aberration root-mean-square (RMS), coma, and spherical aberration, using a standardized pupil diameter of 6.0 mm. Measurements were obtained preoperatively and at the 6-month postoperative visit using Hartmann–Shack or Scheimpflug-based aberrometry systems, depending on platform availability.

Ocular surface status was evaluated using the Ocular Surface Disease Index (OSDI) questionnaire at 6 months, 1 year, and 1.5 years postoperatively. Lower OSDI scores indicated fewer dry eye–related symptoms and reduced impact on visual function.

To minimize inter-device variability, higher-order aberration outcomes were primarily analyzed as induced aberrations (ΔHOAs), calculated as the difference between postoperative and preoperative measurements.

### Outcome measures

The primary outcome measure was refractive predictability, defined as the proportion of eyes achieving a postoperative spherical equivalent within ±0.50 diopters of the intended target refraction (plano, 0.00 D) at 6 months, 1 year, and 1.5 years postoperatively.

Secondary outcome measures included refractive accuracy, defined as the proportion of eyes within ±1.00 diopter of the intended target; refractive stability, assessed by longitudinal changes in mean spherical equivalent over time; visual acuity outcomes (UDVA and CDVA) at each follow-up visit; and safety, evaluated by the proportion of eyes gaining or losing one or more lines of CDVA.

Refractive outcomes were reported according to standardized guidelines for refractive surgery, including predictability, stability, and accuracy metrics (31–33).

Additional secondary outcomes included changes in corneal higher-order aberrations, patient-reported ocular surface symptoms assessed by OSDI scores, the incidence of postoperative complications, and the rate of enhancement (retreatment) procedures during follow-up.

Changes in higher-order aberrations were analyzed as induced aberrations (postoperative minus preoperative values) to allow standardized intergroup comparisons.

### Statistical analysis

Statistical analyses were performed using SPSS software version 27.0 (IBM Corp., Armonk, NY, USA). Continuous variables were assessed for normality using the Shapiro–Wilk test and compared using one-way analysis of variance (ANOVA) or the Kruskal–Wallis test, as appropriate. Categorical variables were compared using the chi-square test.

*Post hoc* pairwise comparisons were performed using the Bonferroni correction when applicable. Missing data were handled using complete-case analysis.

Multivariable linear regression analysis was conducted to identify factors independently associated with residual myopia at the 1.5-year follow-up, defined as the absolute magnitude of postoperative myopia (−spherical equivalent). Covariates included surgical technique, age, baseline myopia magnitude, and central corneal thickness. Variables derived from baseline spherical equivalent, such as categorical myopia degree, were not entered simultaneously into the model to avoid multicollinearity.

Higher-order aberration outcomes were compared among surgical techniques using one-way ANOVA, with Bonferroni-adjusted pairwise comparisons performed where appropriate.

Results were reported as regression coefficients with corresponding 95% confidence intervals. A two-sided P value <0.05 was considered statistically significant.

### Ethical approval

The study was conducted in accordance with the tenets of the Declaration of Helsinki. Ethical approval was obtained from the institutional review boards of Ibn Al-Haitham Teaching Eye Hospital (IRB/IAH/2025-122), Imam Hussain Specialized Eye Complex, Faculty of Medicine (IRB/IHC/2025-212), and Al-Hakeem Teaching Hospital (IRB/HTH/2025-118). All patient data were anonymized before analysis, and the requirement for informed consent was waived owing to the retrospective nature of the study.

## Results

### Study population and baseline characteristics

A total of 919 eyes from 919 patients were included in the final analysis, comprising 388 eyes in the SMILE group, 344 eyes in the FS-LASIK group, and 187 eyes in the Trans-PRK group. Baseline demographic, refractive, and corneal characteristics are summarized in **Table 1**.

**Table 1:** Baseline characteristics of the study population according to surgical technique.

Characteristics	SMILE (n = 388)	FS-LASIK (n = 344)	Trans-PRK (n = 187)	*P-*value
Age (years)	28.48 ± 3.92	28.23 ± 4.05	27.96 ± 3.99	0.324
Sex (male/female), n (%)	214/174 (55.2/44.8)	186/158 (54.1/45.9)	101/86 (54.0/46.0)	0.918
Sphere (D)	−4.94 ± 1.79	−4.95 ± 1.75	−4.67 ± 1.86	0.185
Cylinder (D)	0.78 ± 0.49	0.75 ± 0.46	0.75 ± 0.45	0.643
Spherical equivalent (D)	−4.55 ± 1.78	−4.57 ± 1.72	−4.30 ± 1.86	0.197
LogMAR CDVA	0.00 ± 0.05	0.00 ± 0.05	0.00 ± 0.05	0.234
Central corneal thickness (µm)	536.30 ± 30.30	534.32 ± 30.28	535.37 ± 27.74	0.670
Mean keratometry (D)	43.50 ± 1.25	43.48 ± 1.28	43.50 ± 1.24	0.663
Degree of myopia, n (%)	Mild 112 (28.9) Moderate 186 (47.9) Severe 90 (23.2)	Mild 101 (29.4) Moderate 163 (47.4) Severe 80 (23.3)	Mild 55 (29.4) Moderate 87 (46.5) Severe 45 (24.1)	0.032

The three groups were comparable in terms of age, sex distribution, preoperative spherical equivalent (SE), corrected distance visual acuity (CDVA), central corneal thickness, and mean keratometry (all *P* > 0.05). Although minor differences in the distribution of myopia severity were observed among surgical techniques, no clinically meaningful differences in baseline refractive or corneal parameters were identified.

### Refractive outcomes and visual acuity

Postoperative refractive and visual outcomes at 6 months, 1 year, and 1.5 years are presented in **Tables 2**–**4**.

**Table 2:** Refractive outcomes among SMILE, FS-LASIK, and trans-PRK at 6-month follow-up.

Outcomes	SMILE (n = 388)	FS-LASIK (n = 344)	Trans-PRK (n = 187)	P value
Spherical equivalent (D)	−0.00 ± 0.32	−0.06 ± 0.37	−0.27 ± 0.40	< 0.001
UDVA (LogMAR)	0.06 ± 0.07	0.08 ± 0.08	0.09 ± 0.08	< 0.001
CDVA (LogMAR)	−0.02 ± 0.03	−0.02 ± 0.03	−0.02 ± 0.03	0.364
Eyes within ±0.50 D, n (%)	339 (87.4)	288 (83.7)	127 (67.9)	< 0.001
Eyes within ±1.00 D, n (%)	388 (100.0)	340 (98.8)	180 (96.3)	< 0.001

**Table 3:** Refractive outcomes among SMILE, FS-LASIK, and trans-PRK at 1-year follow-up.

Outcomes	SMILE (n = 388)	FS-LASIK (n = 344)	Trans-PRK (n = 187)	P value
Spherical equivalent (D)	-0.06 ± 0.35	-0.14 ± 0.37	-0.45 ± 0.42	< 0.001
UDVA (LogMAR)	0.08 ± 0.07	0.09 ± 0.08	0.14 ± 0.09	< 0.001
CDVA (LogMAR)	-0.02 ± 0.03	-0.02 ± 0.03	-0.00 ± 0.03	< 0.001
Eyes within ±0.50 D, n (%)	330 (85.1)	274 (79.7)	98 (52.4)	< 0.001
Eyes within ±1.00 D, n (%)	386 (99.5)	338 (98.3)	172 (92.0)	< 0.001

**Table 4:** Refractive outcomes among SMILE, FS-LASIK, and trans-PRK at 1.5-year follow-up.

Outcomes	SMILE (n = 388)	FS-LASIK (n = 344)	Trans-PRK (n = 187)	P value
Spherical equivalent (D)	-0.12 ± 0.37	-0.28 ± 0.39	-0.74 ± 0.43	< 0.001
UDVA (LogMAR)	0.10 ± 0.08	0.12 ± 0.08	0.19 ± 0.09	< 0.001
CDVA (LogMAR)	-0.02 ± 0.03	-0.02 ± 0.03	0.01 ± 0.03	< 0.001
Eyes within ±0.50 D, n (%)	311 (80.2)	239 (69.5)	55 (29.4)	< 0.001
Eyes within ±1.00 D, n (%)	385 (99.2)	334 (97.1)	132 (70.6)	< 0.001

At the 6-month follow-up, significant intergroup differences in postoperative SE were observed (*P* < 0.001). Eyes treated with SMILE achieved refractive outcomes closest to emmetropia, followed by FS-LASIK, whereas Trans-PRK demonstrated greater residual myopia. Uncorrected distance visual acuity (UDVA) was significantly better in the SMILE group than in the FS-LASIK and Trans-PRK groups (*P* < 0.001), whereas CDVA did not differ significantly among groups.

**Figure 1:**
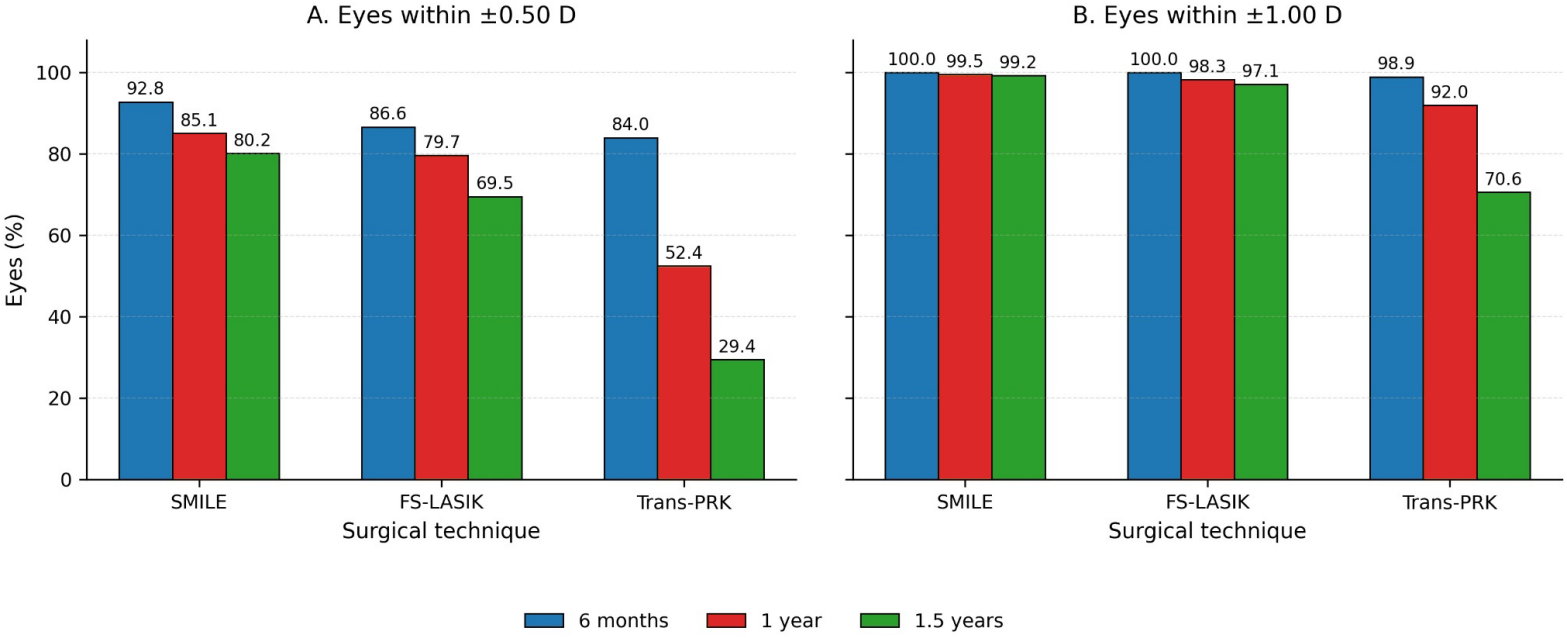
Grouped bar charts comparing SMILE, FS-LASIK, and Trans-PRK for myopic correction at 6 months, 1 year, and 1.5 years, showing **(A)** the percentage of eyes within ±0.50 D and **(B)** the percentage of eyes within ±1.00 D.

Refractive predictability is illustrated in **Figure 1**. The proportion of eyes within ±0.50 D and ±1.00 D of the intended target refraction was consistently highest in the SMILE group across all postoperative time points, followed by FS-LASIK, whereas Trans-PRK demonstrated significantly lower predictability (*P* < 0.001).

At the 1-year follow-up, these intergroup differences persisted. Mean postoperative SE remained closest to emmetropia in the SMILE group, with FS-LASIK demonstrating intermediate outcomes and Trans-PRK showing greater residual myopia (*P* < 0.001). A modest decline in refractive predictability was observed across all techniques, particularly following Trans-PRK.

At 1.5 years, progressive myopic regression was evident in all three groups; however, the magnitude of regression differed significantly by surgical technique (*P* < 0.001). SMILE maintained the most stable refractive outcomes, whereas Trans-PRK exhibited the greatest degree of regression.

**Figure 2:**
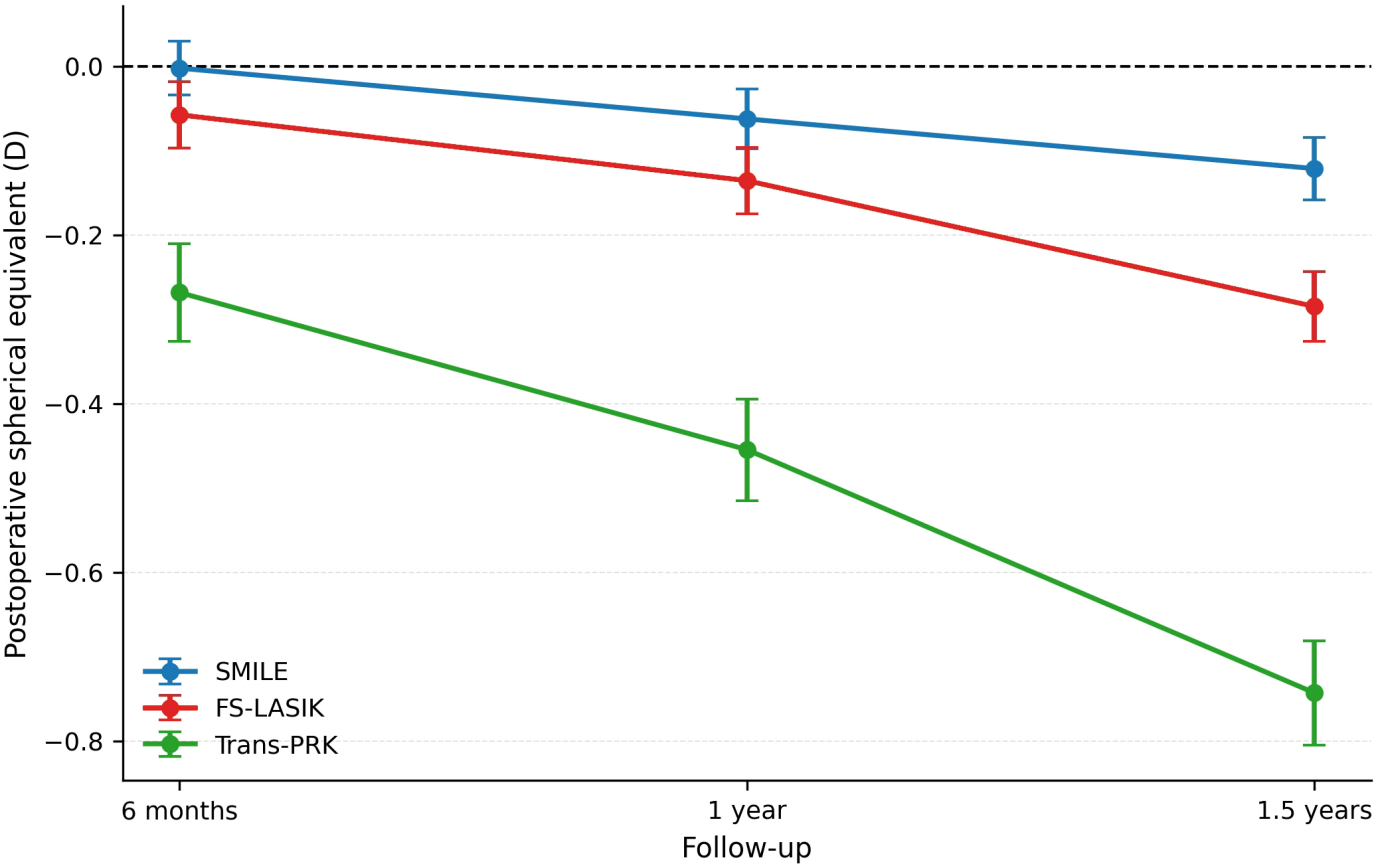
Changes in postoperative spherical equivalent (diopters) over time for SMILE, FS-LASIK, and Trans-PRK at 6 months, 1 year, and 1.5 years.

Refractive stability over time is illustrated in **Figure 2**. All techniques demonstrated a postoperative myopic shift followed by varying degrees of regression. SMILE showed the greatest refractive stability with minimal change in mean SE over time, whereas Trans-PRK exhibited more pronounced myopic regression at longer follow-up intervals.

### Refractive outcomes stratified by degree of myopia

Refractive outcomes stratified by baseline myopia severity are shown in **Table 5**. Across all degrees of myopia, SMILE consistently demonstrated the smallest residual refractive error at each postoperative interval.

**Table 5:** Mean spherical equivalent changes according to the degree of myopia and the surgical technique.

Degree of myopia	Follow-up	SMILE Mean ± SD	FS-LASIK Mean ± SD	Trans-PRK Mean ± SD	P value
Mild	Baseline	−2.1 ± 0.6	−2.0 ± 0.7	−2.0 ± 0.7	0.42
	6 months	−0.05 ± 0.15	−0.10 ± 0.18	−0.20 ± 0.22	<0.001
	1 year	−0.08 ± 0.12	−0.15 ± 0.16	−0.30 ± 0.25	<0.001
	1.5 years	−0.10 ± 0.14	−0.18 ± 0.20	−0.35 ± 0.28	<0.001
Moderate	Baseline	−4.6 ± 0.8	−4.5 ± 0.8	−4.4 ± 0.9	0.31
	6 months	−0.10 ± 0.20	−0.18 ± 0.25	−0.45 ± 0.35	<0.001
	1 year	−0.15 ± 0.22	−0.30 ± 0.28	−0.65 ± 0.40	<0.001
	1.5 years	−0.18 ± 0.25	−0.35 ± 0.30	−0.80 ± 0.45	<0.001
Severe	Baseline	−7.2 ± 0.9	−7.1 ± 0.9	−7.3 ± 1.0	0.27
	6 months	−0.45 ± 0.30	−0.55 ± 0.35	−1.20 ± 0.45	<0.001
	1 year	−0.60 ± 0.35	−0.80 ± 0.40	−1.50 ± 0.55	<0.001
	1.5 years	−0.70 ± 0.40	−0.95 ± 0.45	−1.80 ± 0.60	<0.001

In eyes with mild and moderate myopia, all three techniques achieved satisfactory early refractive correction; however, greater regression over time was observed following Trans-PRK. In eyes with high myopia, intergroup differences were more pronounced, with Trans-PRK exhibiting substantially higher residual myopia at all follow-up time points (*P* < 0.001).

### Refractive accuracy (attempted vs achieved)

**Figure 3:**
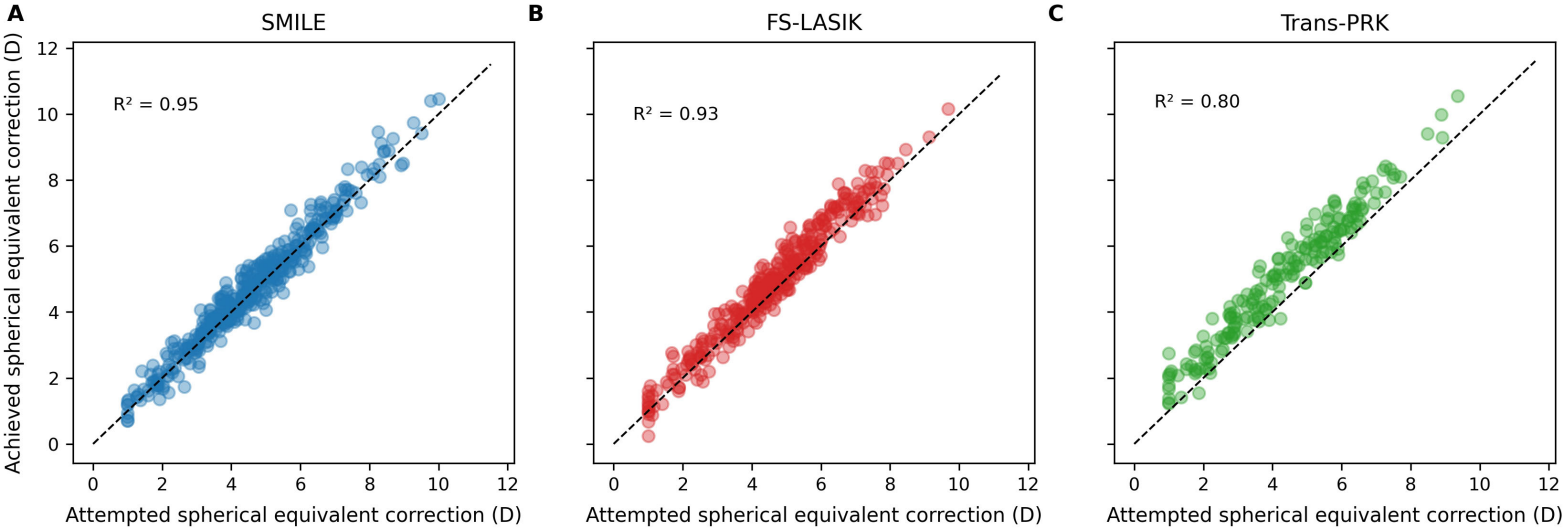
Scatter plots showing the relationship between attempted and achieved spherical equivalent correction at 1.5 years for **(A)** SMILE, **(B)** FS-LASIK, and **(C)** Trans-PRK.

The relationship between attempted and achieved spherical equivalent correction at the 1.5-year follow-up is illustrated in **Figure 3**. A strong linear correlation was observed across all surgical techniques, indicating good overall refractive accuracy.

Among the three procedures, SMILE demonstrated the highest accuracy, as reflected by the strongest correlation (highest R²) and the closest clustering of data points along the line of identity. FS-LASIK showed slightly greater dispersion, whereas Trans-PRK exhibited the greatest variability, indicating reduced precision in achieved refractive outcomes.

### Factors associated with residual refractive error

Results of the multivariable linear regression analysis for residual spherical equivalent (SE) at the 1.5-year follow-up are presented in **Table 6**.

**Table 6:** Multivariable linear regression analysis for residual spherical equivalent at 1.5-year follow-up.

Variable	β coefficient	95% CI	P value
Surgical technique			
FS-LASIK vs SMILE	-0.16	-0.22 to -0.11	<0.001
Trans-PRK vs SMILE	-0.63	-0.70 to -0.56	<0.001
Age (per year)	0.00	-0.00 to 0.01	0.584
Baseline spherical equivalent (per D)	-0.01	-0.04 to 0.02	0.522
Central corneal thickness (per 10 µm)	0.01	-0.00 to 0.01	0.149
Degree of myopia			
Moderate vs Mild	-0.09	-0.19 to 0.02	0.100
Severe vs Mild	-0.17	-0.34 to 0.01	0.071

After adjustment for baseline covariates, surgical technique was independently associated with residual refractive error. Compared with SMILE, FS-LASIK was associated with a significantly more myopic residual SE (β = −0.16, *P* < 0.001), whereas Trans-PRK demonstrated the strongest association with residual myopia (β = −0.63, *P* < 0.001).

Baseline spherical equivalent, age, and central corneal thickness were not independently associated with residual refractive error after multivariable adjustment. Although moderate and severe myopia showed a trend toward greater residual myopia compared with mild myopia, these associations did not reach statistical significance.

### Visual quality outcomes

**Figure 4:**
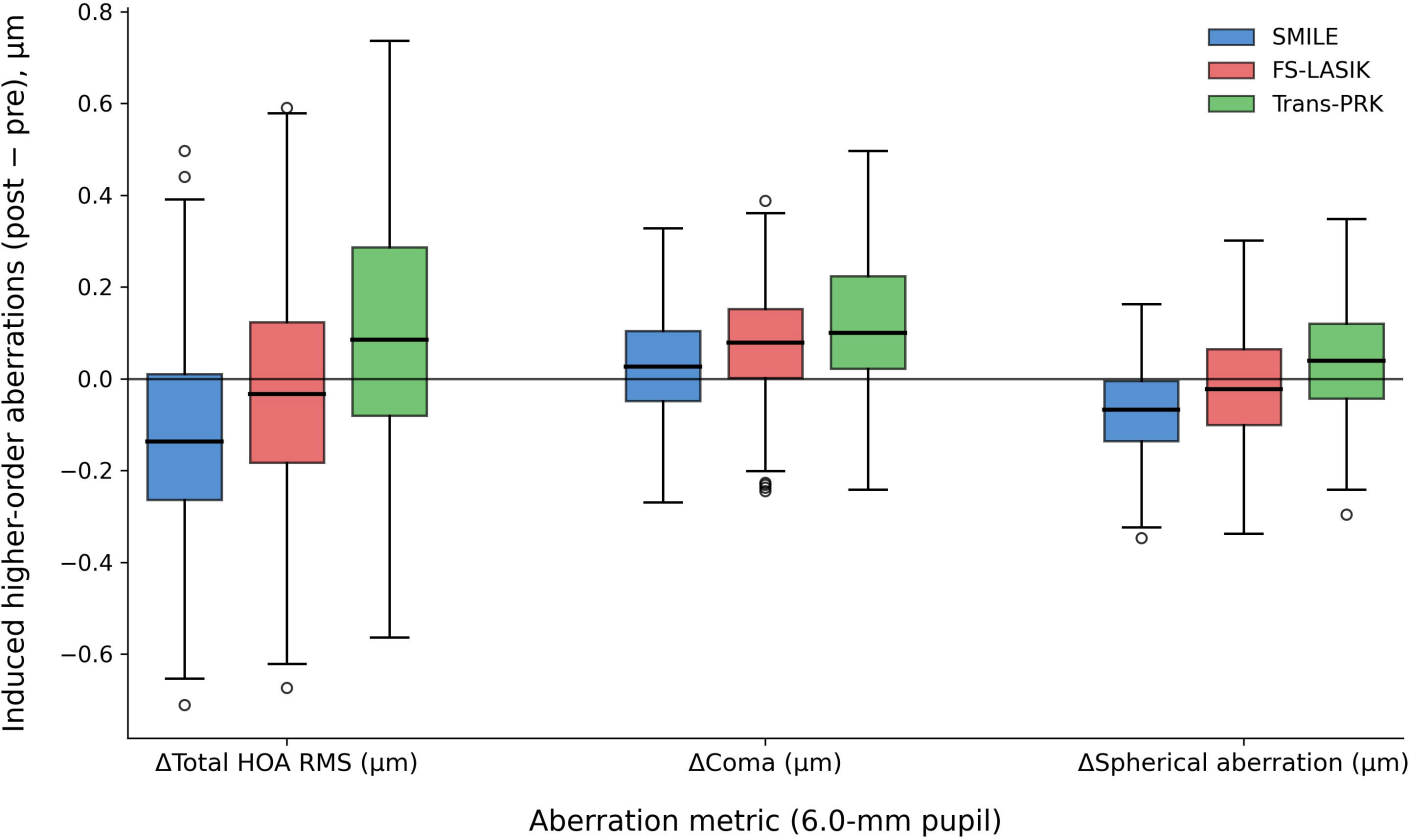
Comparison of induced corneal higher-order aberrations (total HOA RMS, coma, and spherical aberration) among SMILE, FS-LASIK, and Trans-PRK at 6 months postoperatively.

Changes in corneal higher-order aberrations (HOAs) at the 6-month follow-up are shown in **Figure 4**.

Induced HOAs (postoperative minus preoperative values) differed significantly among surgical techniques. SMILE was associated with the lowest induction of total HOA root-mean-square, coma, and spherical aberration, whereas Trans-PRK demonstrated the greatest increase in aberrations. FS-LASIK exhibited intermediate HOA induction. The horizontal reference line at zero indicates no change from baseline.

### Ocular surface outcomes

**Figure 5:**
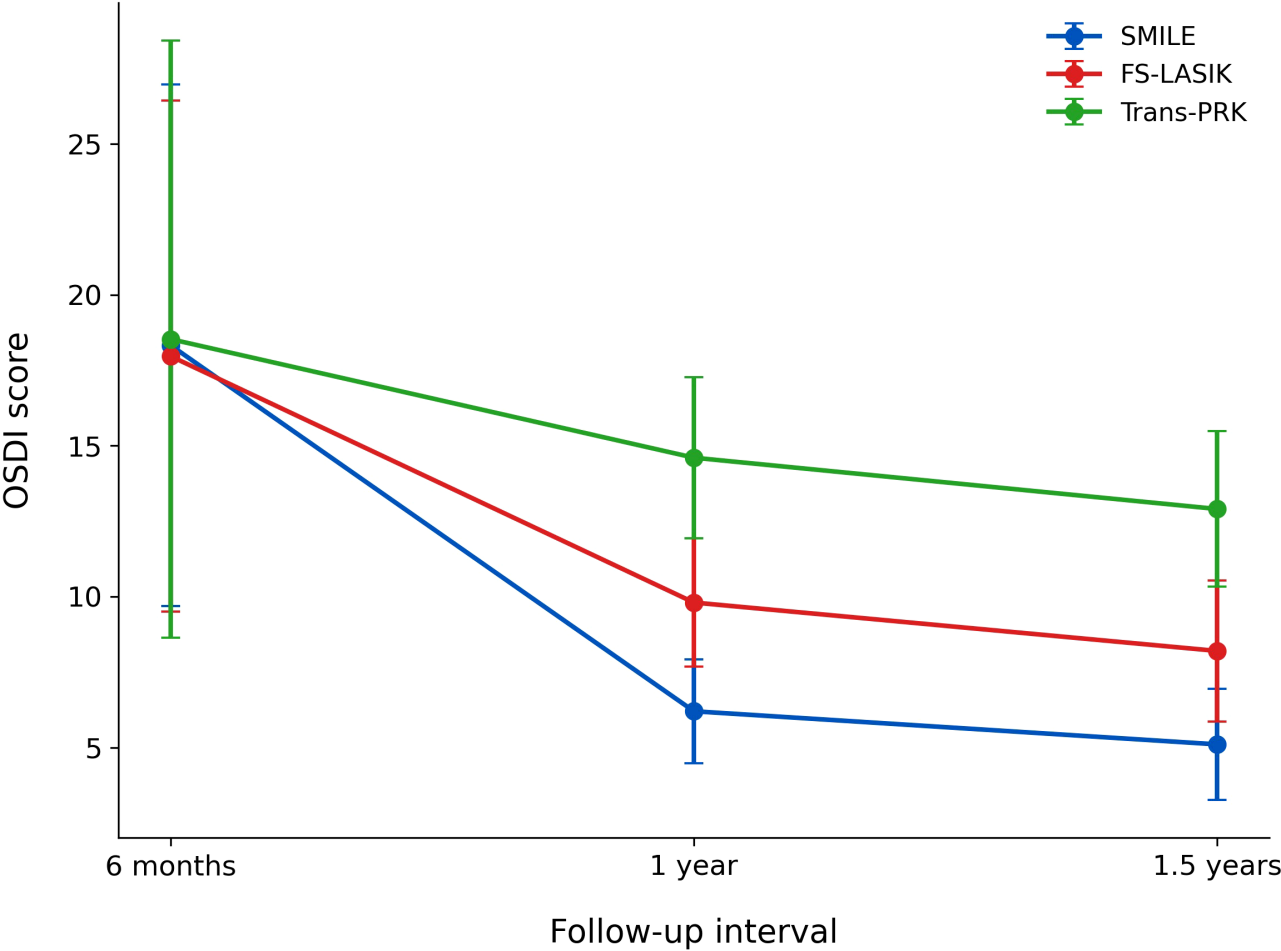
Changes in Ocular Surface Disease Index (OSDI) scores over time for SMILE, FS-LASIK, and Trans-PRK at 6 months, 1 year, and 1.5 years postoperatively.

Longitudinal changes in Ocular Surface Disease Index (OSDI) scores are presented in **Figure 5**.

At all postoperative time points, OSDI scores were lowest after SMILE, intermediate after FS-LASIK, and highest after Trans-PRK. OSDI scores improved progressively over time across all three groups; however, intergroup differences persisted throughout follow-up, indicating more favorable ocular surface outcomes following flapless refractive techniques.

### Safety and complications

All three surgical techniques demonstrated a high safety profile throughout the follow-up period. Loss of one or more lines of corrected distance visual acuity (CDVA) was uncommon at both 6 months and 1.5 years and did not differ meaningfully among the three procedures (**Table 7**).

**Table 7:** Safety, enhancements, and postoperative complications by procedure.

Outcome	SMILE (n=388)	FS-LASIK (n=344)	Trans-PRK (n=187)
Safety (CDVA lines)			
Gain ≥1 CDVA line (6 months)	33 (8.5%)	44 (12.8%)	15 (8.0%)
Loss ≥1 CDVA line (6 months)	8 (2.1%)	5 (1.5%)	2 (1.1%)
Gain ≥1 CDVA line (1.5 years)	33 (8.5%)	44 (12.8%)	6 (3.2%)
Loss ≥1 CDVA line (1.5 years)	8 (2.1%)	5 (1.5%)	9 (4.8%)
Enhancement			
Enhancement by 12 months	2 (0.5%)	3 (0.9%)	6 (3.2%)
Complications			
Haze (3 months)	0 (0.0%)	0 (0.0%)	6 (3.2%)
Epithelial ingrowth	0 (0.0%)	2 (0.6%)	0 (0.0%)
Diffuse lamellar keratitis (DLK)	0 (0.0%)	5 (1.5%)	0 (0.0%)

At the 6-month follow-up, loss of ≥1 CDVA line occurred in 2.1% of eyes after SMILE, 1.5% after FS-LASIK, and 1.1% after Trans-PRK. At 1.5 years, the incidence of CDVA loss remained low across all groups, although a higher proportion was observed after Trans-PRK (4.8%) than after SMILE (2.1%) and FS-LASIK (1.5%).

Enhancement procedures within 12 months were infrequent overall, occurring most commonly after Trans-PRK (3.2%), followed by FS-LASIK (0.9%) and SMILE (0.5%).

Postoperative complications were rare. Transient corneal haze was observed exclusively in the Trans-PRK group (3.2% at 3 months) and resolved without visually significant sequelae. Diffuse lamellar keratitis (DLK) and epithelial ingrowth were observed only in the FS-LASIK group, occurring in 1.5% and 0.6% of eyes, respectively. No cases of corneal ectasia or other sight-threatening complications were identified during follow-up.

## Discussion

This multicenter retrospective cohort study compared refractive outcomes, visual quality, ocular surface status, and safety profiles of SMILE, FS-LASIK, and Trans-PRK for myopia correction in a large Iraqi population. The findings demonstrate procedure-dependent differences in refractive predictability, long-term stability, induction of higher-order aberrations, ocular surface symptoms, and enhancement rates over a follow-up period of up to 1.5 years.

The present study demonstrated that SMILE achieved the most predictable and stable refractive outcomes across all postoperative time points. Eyes treated with SMILE consistently showed postoperative spherical equivalent values closest to emmetropia, narrower distributions of residual refractive error, and reduced long-term myopic regression compared with those treated with FS-LASIK and Trans-PRK. These findings are consistent with multiple long-term comparative studies reporting superior refractive stability following lenticule-based refractive correction, particularly in eyes with moderate-to-high myopia (18, 20, 34, 35).

In contrast, Trans-PRK was associated with greater residual myopia and more pronounced refractive regression, especially in eyes with higher baseline myopia. This observation aligns with previous reports suggesting that surface ablation techniques are more susceptible to postoperative stromal wound healing responses and epithelial remodeling, which may contribute to refractive drift over time (36–39). FS-LASIK demonstrated intermediate refractive stability, consistent with prior studies emphasizing the importance of flap thickness, preservation of the residual stromal bed, and optical zone planning in achieving stable long-term outcomes (10, 35, 40).

Multivariable regression analysis identified surgical technique as the primary independent predictor of residual refractive error at the 1.5-year follow-up. Compared with SMILE, both FS-LASIK and Trans-PRK were associated with significantly greater residual myopic spherical equivalent, with the strongest association observed for Trans-PRK. After adjustment, baseline spherical equivalent, age, and central corneal thickness were not independently associated with residual refractive error.

These findings support prior evidence indicating that procedure-related biomechanical and biological factors may exert a greater influence on long-term refractive stability than baseline anatomical parameters alone (20, 28, 41). Although increasing myopia severity was associated with greater residual myopia, this association did not reach statistical significance after multivariable adjustment, highlighting the dominant role of surgical technique in determining refractive outcomes.

Assessment of induced corneal higher-order aberrations demonstrated lower induction of total HOA root-mean-square, coma, and spherical aberration following SMILE compared with FS-LASIK and Trans-PRK. Preservation of the anterior corneal lamellae and avoidance of flap creation in SMILE may contribute to reduced biomechanical disruption and improved postoperative optical quality (17, 19, 42, 43).

FS-LASIK exhibited intermediate HOA induction, whereas Trans-PRK showed the greatest increase in aberrations. These findings are in agreement with several comparative studies reporting more favorable HOA profiles following SMILE, although the literature remains heterogeneous (34, 42, 44). Differences in pupil size standardization, centration strategies, measurement platforms, and ablation algorithms may partially account for variability across studies. The use of induced (postoperative minus preoperative) HOA values in the present study strengthens the interpretability of the visual quality analysis.

Longitudinal analysis of Ocular Surface Disease Index (OSDI) scores demonstrated consistently lower symptom scores following SMILE, with FS-LASIK showing intermediate outcomes and Trans-PRK associated with higher scores throughout follow-up. Although OSDI scores improved over time across all three procedures, intergroup differences persisted, indicating sustained ocular surface advantages with flapless refractive techniques.

These findings are consistent with prior studies and meta-analyses reporting reduced postoperative dry eye symptoms following SMILE compared with FS-LASIK, likely related to better preservation of corneal innervation and reduced disruption of the ocular surface (45–47). Environmental factors such as high ambient temperatures, low humidity, and increased exposure to airborne particulates characteristic of the Middle Eastern region may further influence postoperative ocular surface outcomes. They may partly explain the persistence of symptoms observed following flap-based or surface ablation procedures (48, 49).

All three refractive procedures demonstrated a high safety profile, with low rates of CDVA loss and no sight-threatening complications observed during follow-up. Loss of one or more lines of CDVA was uncommon at both 6 months and 1.5 years across all techniques, consistent with large registry-based and comparative studies reporting high safety indices for contemporary laser refractive surgery (50–52).

Enhancement procedures were infrequent overall, but most commonly occurred after Trans-PRK, reflecting its lower refractive predictability and greater susceptibility to regression. Procedure-specific complications followed expected patterns: transient corneal haze occurred exclusively after Trans-PRK, whereas diffuse lamellar keratitis and epithelial ingrowth were observed only following FS-LASIK. Importantly, these events were rare and resolved without permanent visual impairment.

The findings of this study suggest that SMILE offers a favorable balance of refractive accuracy, long-term stability, optical quality, ocular surface comfort, and safety for myopia correction in Iraqi patients. FS-LASIK remains a highly effective alternative with excellent visual outcomes. At the same time, Trans-PRK continues to play an important role in selected cases, particularly in eyes with thin corneas or contraindications to flap-based surgery. Careful patient selection and counselling remain essential, especially for individuals with higher degrees of myopia.

Strengths of the present study include its large sample size, multicenter design, inclusion of three widely used contemporary refractive techniques, and comprehensive evaluation of refractive, visual quality, ocular surface, and safety outcomes. Importantly, the study provides population-specific data from Iraq, a region underrepresented in refractive surgery research.

This study has several limitations. First, its retrospective design, which inherently yields lower-level evidence than randomized controlled trials, along with the non-randomized allocation of surgical techniques, may introduce selection bias despite adjustment for baseline covariates. In addition, variability in surgical platforms and surgeon-specific nomograms across centers may have influenced the observed outcomes. However, this heterogeneity reflects real-world clinical practice rather than a controlled experimental setting, and the consistency of findings across analyses of predictability, stability, and accuracy suggests that the results are unlikely to be attributable solely to nomogram-related factors.

Furthermore, the inclusion of both wavefront-optimized and wavefront-guided ablation profiles within the FS-LASIK group may have introduced heterogeneity in higher-order aberration outcomes, given that these ablation strategies have been shown to differentially affect postoperative visual quality and aberration profiles (53, 54). Visual quality assessment was also limited by evaluating higher-order aberrations at a single postoperative time point and by the absence of objective tear film measurements, despite longitudinal assessment using OSDI.

Additionally, endothelial cell density and hexagonality were not evaluated, as specular microscopy data were not consistently available across all participating centers. Finally, although no cases of postoperative ectasia were observed, the study was not specifically designed to evaluate ectasia risk or the predictive role of preoperative corneal parameters, including keratometry.

## Conclusion

In this large multicenter Iraqi cohort, contemporary laser refractive surgery techniques demonstrated high efficacy and safety for myopia correction. Among the three procedures evaluated, small incision lenticule extraction (SMILE) achieved the highest refractive predictability and long-term stability, with lower residual refractive error, reduced induction of higher-order aberrations, and more favorable patient-reported ocular surface outcomes over 1.5 years of follow-up.

Femtosecond laser–assisted LASIK (FS-LASIK) demonstrated intermediate refractive stability, excellent visual acuity outcomes, and a low complication rate, making it a reliable and effective option for appropriately selected patients. Transepithelial photorefractive keratectomy (Trans-PRK) was effective in achieving myopic correction. Still, it was associated with greater refractive regression, higher enhancement rates, and less favorable ocular surface outcomes, particularly in eyes with higher degrees of myopia.

Surgical technique emerged as the primary determinant of long-term refractive outcomes, underscoring intrinsic differences among lenticule-based, flap-based, and surface ablation procedures that extend beyond baseline refractive characteristics alone. These findings provide population-specific evidence from Iraq and support careful selection of procedures and patient counselling to optimize refractive accuracy, visual quality, and postoperative comfort.

## Data Availability

The datasets presented in this study can be found in online repositories. The names of the repository/repositories and accession number(s) can be found in the article/supplementary material.
